# Climate Change Perceptual Awareness, Climate-Related Anxiety, and Perceived Impacts of Climate Change Among University Students in Jordan: Findings from a Multi-University Cross-Sectional Study

**DOI:** 10.3390/ijerph23050649

**Published:** 2026-05-13

**Authors:** Ala’a B. Al-Tammemi, Hindya O. Al-Maqableh, Mohammad Aljarrah, Sami A. Alhallaq, Ahmad W. A. AlKhyat, Hanan Hasan, Dua’a Al Tamimi, Malak T. Alnatsheh, Hedaya R. Hina, Eman Mohammad Qudah, Baraa Joudeh, Muayyad Islam Abusido, Mus’ab Banat, Abrar Omari, Dana Jamal Suboh, Sahar H. AlAhmad, Redab Al-Ghawanmeh, Dalia Kashef Zayed, Salam Momani, Haitham Khatatbeh, Ibrahim Ayasreh, Rabaa Y. Athamneh, Moawiah Khatatbeh, Muna Barakat, Fayez Abdulla, Mohammad Al-Qudah, Tareq L. Mukattash

**Affiliations:** 1 Jordan Center for Disease Control, Amman 11183, Jordans.momani@jcdc.gov.jo (S.M.);; 2 GeoHealth Hub—MENA, Jordan University of Science and Technology, Irbid 22110, Jordan; 3 Industrial Engineering Department, Faculty of Engineering, The Hashemite University, Zarqa 13133, Jordan; 4 Department of Internal Medicine, Division of Pulmonology, King Hussein Medical City, Jordanian Royal Medical Services, Amman 11733, Jordan; 5 Al-Waleed Medical Laboratories, Amman 11937, Jordan; 6 Department of Community Health Nursing, Princess Muna College of Nursing, Mutah University, Amman 11733, Jordan; 7 Community Health Nursing Department, School of Nursing, The University of Jordan, Amman 11942, Jordan; 8 Al-Bashir Hospitals, Ministry of Health, Amman 11151, Jordan; 9 Faculty of Nursing, German Jordanian University, Amman 11180, Jordan; 10 English Department, Faculty of Arts, Yarmouk University, Irbid 21163, Jordan; 11 Faculty of Pharmacy, Amman Arab University, Amman 11953, Jordan; baraajoudeh966@gmail.com; 12 Aqaba Medical Sciences University, Aqaba 77110, Jordan; 13 Prince Hamza Hospital, Amman 11947, Jordan; 14 Faculty of Medicine, Al-Balqa Applied University, Salt 19117, Jordan; 15 Department of Digital Communication and Media, Faculty of Arts, The University of Jordan, Amman 11942, Jordan; 16 Department of Pediatrics, Faculty of Medicine, The Hashemite University, Zarqa 13133, Jordan; 17 Faculty of Nursing, Yarmouk University, Irbid 21163, Jordan; 18 Department of Adult Nursing, Faculty of Nursing, Jerash University, Jerash 26150, Jordan; 19 Department of Medical Laboratory Sciences, Faculty of Allied Medical Sciences, Zarqa University, Zarqa 13132, Jordan; 20 Department of Basic Medical Sciences, Faculty of Medicine, Yarmouk University, Irbid 21163, Jordan; 21 Department of Clinical Pharmacy and Therapeutics, Faculty of Pharmacy, Applied Science Private University, Amman 11937, Jordan; 22 Civil Engineering Department, Jordan University of Science and Technology, Irbid 22110, Jordan; fabdulla@just.edu.jo; 23 Department of Microbiology and Pathology, Faculty of Medicine, The Hashemite University, Zarqa 13133, Jordan; 24 Department of Pathology and Microbiology, Faculty of Medicine, Jordan University of Science and Technology, Irbid 22110, Jordan; 25 Department of Clinical Pharmacy, Faculty of Pharmacy, Jordan University of Science and Technology, Irbid 22110, Jordan

**Keywords:** climate change, climate awareness, Jordan, university students, MENA, CCPAS, CCAS, eco-anxiety, climate policy, climate anxiety

## Abstract

**Highlights:**

**Public health relevance—How does this work relate to a public health issue?**

- Climate change is an escalating public health challenge with implications for physical and mental wellbeing, as well as population resilience.
- This study addresses climate change awareness and anxiety among university students in Jordan, an important population for future public health action.

**Public health significance—Why is this work of significance to public health?**

- The findings reveal high general awareness of climate change but limited awareness of Jordan’s national climate change policy.
- The study also identifies measurable differences in climate change anxiety and perceptual awareness across population sub-groups.

**Public health implications—What are the key implications or messages for practitioners, policy makers and/or researchers in public health?**

- Climate-health communication and education should be strengthened within universities and youth-focused public health programs.
- Public health policy and research should address both climate literacy and the psychosocial burden of climate change in vulnerable young populations.

**Abstract:**

**Background**: Jordan is increasingly recognized as a climate-vulnerable setting in the region, yet evidence on the psychosocial dimensions of climate change among young adults remains limited. Led by the Jordan Center for Disease Control, this study assessed climate change perceptual awareness and climate-related anxiety among university students and explored perceptions of climate impacts at global and national levels. **Methods**: In a cross-sectional survey conducted between April and May 2025, 1305 students were recruited from universities across Jordan using a questionnaire incorporating the 15-item Climate Change Perceptual Awareness Scale and the 13-item Climate Change Anxiety Scale. **Results**: Awareness of climate change and global warming was high (87% and 96%, respectively), yet only 23% were familiar with Jordan’s National Climate Change Policy 2022–2050. In regression analyses, higher climate anxiety was associated with female sex, married status, larger household size, diagnosed mental health conditions, and central-region university enrolment. Higher perceptual awareness was associated with female sex, older age, and the recognition of multidimensional climate impacts. Students identified heatwaves, drought, and forest fires as principal environmental threats, and respiratory and heat-related illnesses as foremost health concerns. **Conclusions**: Our findings position climate change as not only an environmental concern, but also as an educational, psychological, and public health priority. To support effective adaptation and resilience, climate awareness must be translated into informed engagement and action. Integrating climate and climate-health education into university curricula, improving youth-responsive communication of national climate strategies, and creating formal pathways for youth participation in climate governance are essential investments in Jordan’s climate resilience, health security, and long-term sustainable development.

## 1. Introduction

Climate change represents one of the most significant public health, environmental, and psychosocial challenges of the 21st century. The Middle East and North Africa (MENA) region is widely recognized as a climate change hotspot, where extreme aridity, high temperatures, and decreasing precipitation amplify vulnerabilities [1]. In addition to the biophysical impacts, climate change in the region is tightly interwoven with complex psychosocial and behavioral dimensions. These dimensions manifest as eco-anxiety, and other mental health issues, while also influencing the adoption of sustainability practices and community-level adaptation measures [2,3]. Recent scholarly efforts have shifted from exclusive emphasis on the physical manifestations of climate change to multidisciplinary inquiries that incorporate human perceptions, emotional responses, and adaptive behaviors. Such approaches underscore the necessity to understand how cognitive factors, direct and indirect exposure to extreme weather events, and socio-political contexts shape risk assessments and behavioral responses [4,5]. Empirical evidence suggests that although people in many MENA countries recognize climate change as a general threat, they often underestimate or misunderstand its underlying causes, frequently conflating climate change with other environmental issues [6]. Together, these observations point to a critical gap between broad climate awareness and deeper psychosocial and cognitive engagement with climate risk, a gap that demands systematic investigation, particularly in vulnerable regions such as the MENA, where the psychosocial burden of climate change remains poorly understood.

Eco-anxiety, a state of chronic fear specifically related to environmental destruction and climate catastrophe, has emerged as a prevalent mental health concern throughout the MENA region. The rapid pace of climate change, coupled with recurring extreme weather events and environmental decline, has been directly linked to increased levels of anxiety, depression, and post-traumatic stress in vulnerable populations [3,7]. The phenomenon of eco-anxiety is not isolated; rather, it interrelates with broader patterns of mental health degradation triggered by environmental stressors. Additionally, perceptual awareness of climate change refers to a person’s subjective awareness and sensory-perceptual recognition of the everyday effects of climate change [8]. This concept is distinguished from general attitudes or beliefs because it focuses more on what people perceive and experience, rather than only what they think or believe about climate change.

Our study draws on Lazarus and Folkman’s Transactional Model of Stress and Coping, which emphasizes that psychological distress is shaped not only by exposure to an environmental stressor, but also by individuals’ cognitive appraisal of that stressor as threatening and by their perceived capacity to manage or cope with it [9]. Applied to climate change, this model suggests a sequential pathway in which individuals who are more perceptually aware of climate change in their daily environment are more likely to appraise it as a serious personal threat, and this appraisal, in turn, elevates psychological distress [10,11]. This framework is complemented by Protection Motivation Theory [12], which distinguishes between threat appraisal, encompassing perceived severity and personal vulnerability, and coping appraisal, encompassing perceived response efficacy, self-efficacy, and the perceived costs of undertaking protective action. In the climate change context, these constructs help explain why perceived threat may translate into anxiety, adaptive engagement, or perceived helplessness depending on individual and collective efficacy beliefs and on whether the costs of action are judged to be manageable. Empirical evidence is broadly consistent with this pathway, showing associations between climate change awareness, exposure to climate-related events, perceived risk, worry, and climate-related anxiety, although the direction of these relationships may be bidirectional and should be interpreted cautiously in cross-sectional studies [13,14]. Sociodemographic and contextual factors, including age, gender, geographic setting, and health vulnerability, may further shape climate change appraisal and its emotional consequences [15].

Building on these appraisal-based frameworks, the climate psychology literature distinguishes between adaptive and maladaptive forms of eco-anxiety. At lower levels, eco-anxiety may sustain alertness to climate risks and motivate pro-environmental engagement, whereas more severe levels are associated with persistent worry, helplessness, and clinically meaningful functional distress [16,17]. It should be noted, however, that empirical support for this distinction remains mixed, and the direction of the relationship between eco-anxiety and pro-environmental behavior is debatable. The proposition is therefore best understood as a working conceptualization that guided the dimensional structure of the anxiety measure used in the present study rather than as a firmly established dose–response relationship. Young people are particularly vulnerable within this broader framework, as their heightened developmental orientation toward the future, characterized by greater investment in long-term outcomes and heightened sensitivity to intergenerational threat, amplifies the subjective weight of climate-related risks. Hickman and colleagues confirmed this vulnerability empirically, demonstrating in a multi-country survey of around 10,000 young people aged 16 to 25 that the majority experienced significant climate-related worry, anxiety, and feelings of powerlessness, though their study attributed this primarily to perceived inadequacy of governmental responses rather than to the developmental mechanism per se [5]. Drawing on the logic of the Social Amplification of Risk Framework, eco-anxiety is understood here as being shaped not only by direct or perceived exposure to climate hazards but also by how institutional and governmental responses to those hazards are perceived, communicated, and interpreted within social and cultural contexts [18].

In Jordan, a middle-income country in the MENA region, climate change is exacerbating pre-existing environmental and socioeconomic vulnerabilities. As one of the most water-stressed countries in the world, Jordan is experiencing declining precipitation and rising evaporation rates that further deplete limited water resources, reduce agricultural productivity, and threaten national food security. Concurrently, rising temperatures and heatwaves are increasing health risks and adding pressure on already strained infrastructure and public health system. These intersecting climate-related pressures are further compounded by rapid population growth and resource depletion, which together constrain the country’s adaptive capacity. Nevertheless, despite Jordan’s pronounced exposure to climate-related risks, the psychosocial dimensions of climate change remain insufficiently examined. This evidence gap hampers the design of adaptation strategies that depend on behavioral and psychosocial integration. The literature increasingly frames climate change as both an environmental and psychosocial threat, with global data indicating rising levels of eco-anxiety, particularly among younger populations [5]. Perceived impacts influence behavioral responses [6], but gaps remain in many countries where research addresses environmental management rather than psychosocial responses.

Jordan is among the world’s youngest nations, with 63% of its population below the age of 30 [19,20]. Jordanian youth already experience substantial psychological distress linked to socioeconomic hardship, and other social stressors [21], yet there is still no systematic evidence on climate-related eco-anxiety in this population, and most research on this phenomenon to date has been conducted among adults rather than youth [22]. Given that university students constitute a large segment of Jordan’s youth, this project sought to comprehensively assess climate change perceptual awareness and climate-related anxiety in this population, to examine associations with both outcomes across various sociodemographic, academic, geographic, and health-related factors, and to examine students’ perceptions of climate change impacts at both global and national levels.

## 2. Materials and Methods

### 2.1. Study Design, Setting, and Study Population

A multi-university cross-sectional survey was conducted in Jordan between April and May 2025 under the leadership of the Jordan Center for Disease Control (JCDC). Multiple higher education institutions across all Jordanian regions were engaged and were formally contacted and onboarded via official letters issued by JCDC, after which focal points at various institutions facilitated local coordination and student outreach. Eligible participants were students aged ≥18 years and drawn from diverse academic stages and fields of study.

### 2.2. Data Collection Tools

Data were collected via an anonymous, self-administered online questionnaire distributed using Google Forms^®^ to students through their respective higher education institutions using a convenience sampling approach. Because the survey invitation was circulated through institutional communication channels and no individual-level invitation log was available, the number of students who received, viewed, or had access to the survey could not be ascertained. Consequently, a response rate could not be calculated.

**Figure 1:**
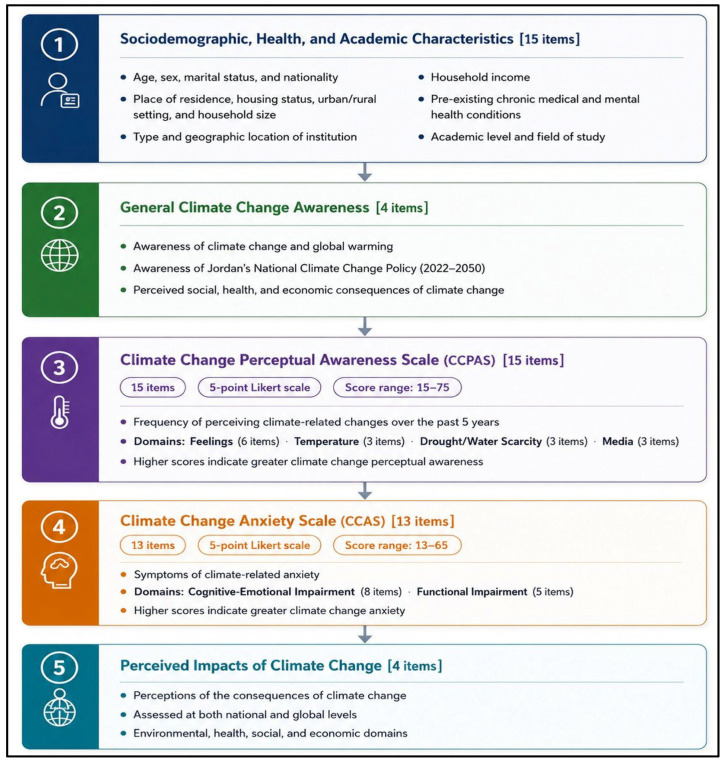
Survey components.

As shown in Figure 1, the survey questionnaire comprised 51 items organized into five sections. It included the following: (i) 15 items on sociodemographic, health, and academic characteristics; (ii) four items assessing general awareness of climate change and global warming; (iii) the 15-item Climate Change Perceptual Awareness Scale (CCPAS); (iv) the 13-item Climate Change Anxiety Scale (CCAS); and (v) four items evaluating students’ perceptions and beliefs regarding the consequences of climate change at global and national levels.

The sociodemographic, health, and academic section captured self-reported characteristics, including the following: age, sex, marital status, nationality, governorate of residence, housing type, residential setting, household size, average monthly household income, presence of chronic medical conditions, presence of chronic mental health conditions, institution type, geographic location of the academic institution, study level, and field of study.

In the general awareness section, students were asked whether they had heard of climate change and global warming, whether they were aware of Jordan’s national climate change policy 2022–2050, and whether they believed climate change has social, health, or economic consequences.

We measured climate change perceptual awareness using the 15-item CCPAS [23], which asked students to report how often they have personally noticed climate-related changes or reactions in the last five years. Items are rated on a five-point Likert scale (1 = Never, 2 = Rarely, 3 = Occasionally, 4 = Frequently, 5 = Very frequently). The 15-item CCPAS comprises four subscales: feelings (six items), temperature (three items), drought/water scarcity (three items), and media (three items). Subscale scores are calculated by summing their designated items. Higher scores indicate greater perceptual awareness in that domain. Also, the total CCPAS score is reported by summing all 15 items (range 15–75), with higher scores indicating greater perceptual awareness and more pro-environmental behavior. The 15-item CCPAS showed excellent internal reliability in its original validation [8,23].

The original English version of the 15-item CCPAS was adapted into Arabic through a systematic process aimed at ensuring semantic clarity and contextual suitability for the target population. First, the instrument was forward-translated from English into Arabic by a bilingual translator who was familiar with the study context. The preliminary Arabic version was then reviewed to ensure conceptual and contextual equivalence with the original English items. Subsequently, a backward translation into English was performed by an independent bilingual translator who had not been involved in the initial translation process. The back-translated version was compared with the original English scale to identify discrepancies in meaning, phrasing, and interpretation, and the Arabic version was revised accordingly. The pre-final Arabic version was then evaluated by a panel of three experts with relevant expertise in public health, research methodology, and bilingual academic communication to assess clarity, cultural appropriateness, relevance, and comprehensibility of the items. Based on experts’ feedback, minor linguistic modifications were introduced to improve wording precision and readability while preserving the intended meaning of the original items. Thereafter, the revised Arabic version was pilot-tested on 30 university students from the target population to assess face validity, clarity, acceptability, and ease of understanding. Pilot-phase feedback indicated that no further linguistic refinements were needed, and the finalized Arabic version was therefore used in the present study.

Overall, the Arabic version of the 15-item CCPAS showed good internal consistency, indicating that the items worked well together as a single measure of climate change perceptual awareness. The overall scale had a Cronbach’s alpha of 0.86, which reflects good reliability. The four domains of the scale also showed acceptable to good reliability: drought and water scarcity had a Cronbach’s alpha of 0.78, media had a Cronbach’s alpha of 0.78, temperature had a Cronbach’s alpha of 0.75, and feelings had a Cronbach’s alpha of 0.84.

The factorial structure for the Arabic translation of the 15-item CCPAS was examined using both exploratory and confirmatory factor analyses. Before conducting factor analysis, the suitability of the item correlation matrix was assessed using the Kaiser–Meyer–Olkin (KMO) measure of sampling adequacy and Bartlett’s test of sphericity. Exploratory factor analysis (EFA) was used to investigate the underlying dimensional structure of the scale. Because CCPAS items use a five-point Likert-type ordinal response format, confirmatory factor analysis (CFA) was conducted using the Diagonally Weighted Least Squares (DWLS) estimator applied to the polychoric correlation matrix, with robust standard errors and Satorra–Bentler mean correction. The Satorra–Bentler mean correction was applied to the chi-square statistic to adjust for non-normality. Robust standard errors were computed using the sandwich estimator. Dimensional invariance across sex was examined using a multi-group configural model.

Model fit was evaluated using the comparative fit index (CFI), Tucker–Lewis index (TLI), root mean square error of approximation (RMSEA), and standardized root mean square residual (SRMR). Conventional interpretive thresholds were applied, whereby CFI and TLI values ≥0.95 were considered indicative of good fit and values ≥0.90 of acceptable fit; RMSEA values ≤0.06 indicated close fit, with values ≤0.08 generally considered acceptable; and SRMR values ≤0.08 were considered indicative of good fit. Given the large sample size (*n* = 1305), the chi-square statistic is expected to be significant regardless of model quality; therefore, it was not used as the primary fit criterion.

The KMO measure of sampling adequacy was 0.882, and Bartlett’s test of sphericity was statistically significant χ^2^(105) = 9381.12, *p* < 0.001, indicating that the inter-item correlations were adequate for examination of latent structure. The model demonstrated acceptable fit with DWLS χ^2^(84) = 4067.12, *p* < 0.001, CFI = 0.931, TLI = 0.914, RMSEA = 0.191, SRMR = 0.146. All standardized factor loadings were statistically significant (*p* < 0.001) and ranged from 0.650 to 0.893, with average variance extracted values (AVE) ≥0.50 across all four subscales (range: 0.577–0.630). Multi-group configural CFA indicated acceptable fit in both male (CFI = 0.944, TLI = 0.930, RMSEA = 0.175, SRMR = 0.137) and female (CFI = 0.928, TLI = 0.910, RMSEA = 0.196, SRMR = 0.151) subsamples, supporting configural invariance of the CCPAS factor structure across sex. The omnibus multi-group configural model produced χ^2^(168) = 4179.69, CFI = 0.932, TLI = 0.915, RMSEA = 0.191, closely replicating the fit profile of the single-group solution.

These findings indicate that the Arabic version of the 15-item CCPAS performed well at the item and subscale level, with strong standardized loadings and acceptable convergent validity. Nevertheless, the four-factor model was not fully supported by global fit indices, particularly RMSEA and SRMR. Therefore, the Arabic translation of the 15-items CCPAS used in our study may be described as showing preliminary psychometric adequacy for use in this population (university students), while further validation in more diverse Arabic-speaking samples is highly warranted.

Climate-related anxiety was assessed using the 13-item Climate Change Anxiety Scale (CCAS) developed by Clayton and Karazsia [24], which captures climate anxiety as a psychological response to climate change. The CCAS has two domains: cognitive–emotional impairment (eight items) and functional impairment (five items). Respondents rate each statement on a five-point Likert scale from 1 = Never to 5 = Almost always. The overall CCAS score ranges from 13 to 65.

An Arabic translation of the CCAS has been validated in Lebanese adults [25], confirming the original two-factor structure (cognitive–emotional and functional impairment), excellent overall scale internal reliability (Cronbach’s alpha = 0.96), and measurement invariance across gender, thus supporting its use in Arabic-speaking populations. Moreover, the availability of an Arabic version is further supported by another study that validated the 13-item CCAS in a sample drawn from five Arab countries, with findings confirming its satisfactory psychometric performance and reinforcing its suitability for assessing climate change-related anxiety in Arab populations [26]. In our study, the Arabic 13-item CCAS had excellent internal reliability with Cronbach’s alpha of 0.93.

In the final section, students evaluated perceived global impacts of climate change across various environmental domains; identified the single most important climate-related health consequence in the Middle East; projected the magnitude of climate change effects over the next 50 years, contrasting anticipated global versus Jordan-specific outcomes; and estimated the impacts of global warming over the next 20 years under a no-action scenario. The items in this section were adopted from a previously published study [27].

### 2.3. Data Analysis

Data from the survey were extracted and exported to Statistical Package for Social Sciences, SPSS^®^ for Windows (IBM Corp. Version 27.0. Armonk, NY, USA) for quality check and analysis. The distributions of CCAS and CCPAS scores were evaluated using histograms, normal Q–Q plots, as well as skewness and kurtosis statistics. As both outcome variables were not normally distributed, descriptive results were presented as median and interquartile range (IQR) rather than mean and standard deviation (SD). Comparisons of CCAS and CCPAS scores across categorical variables were performed using the Mann–Whitney U test or Kruskal–Wallis test, as appropriate. The relationship between CCAS and CCPAS scores was assessed using Spearman’s rank correlation coefficient. In addition, a simple linear regression model was fitted to describe the direction and magnitude of the linear association between CCAS and CCPAS.

Associations between CCPAS and CCAS scores and independent variables were examined using multivariable linear regression. Before model fitting, the assumptions of linear regression were assessed for both outcomes. The distributions of CCAS and CCPAS scores, as well as residuals, were non-normal, and Breusch–Pagan testing indicated heteroskedasticity. Multicollinearity was not considered substantial, with all variance inflation factors (VIFs) below 10 and all tolerance values exceeding 0.1. In view of the observed non-normality of residuals and heteroskedasticity, the primary models were estimated using heteroskedasticity-robust standard errors. Statistical significance was set at *p*-value of less than 0.05.

## 3. Results

### 3.1. Participant Characteristics

A total of 1305 students consented to participate in this national cross-sectional study. The median age of participants was 21 years (IQR: 19–23 years), reflecting a predominantly young adult population. The sample showed a clear predominance of females, who accounted for most participants (*n* = 1020; 78.2%), compared with males. Regarding marital status, most participants were single (*n* = 1169, 89.6%). The sample was predominantly Jordanian nationals (*n* = 1179, 90.3%).

Geographic distribution revealed that more than half of the respondents resided in the central region of Jordan (*n* = 721, 55.3%), followed by the northern region (*n* = 505, 38.7%), and the southern region (*n* = 79, 6.0%). In terms of living arrangements, the majority of students resided in their family homes (*n* = 1070, 82.0%). Urban residents constituted 62.8% (*n* = 819) of the sample. Household characteristics showed that most participants came from larger families, with 62.9% (*n* = 821) reporting five or more household members.

In terms of socioeconomic status, as measured by average monthly income of the family (1 Jordanian Dinar = 1.41 U.S. Dollar), the findings revealed that approximately half of the sample came from lower-income households as the largest proportion of participants (*n* = 641, 49.1%) reported family incomes of ≤600 Jordanian Dinars (≤846 US dollars).

With respect to health status, most participants (*n* = 1108, 84.9%) reported no currently diagnosed chronic medical conditions under treatment. Mental health diagnoses were less prevalent, with only 7.5% (*n* = 98) reporting current mental health conditions. See Table 1 for more details.

Regarding institutional affiliation, most participants (*n* = 909, 69.7%) were enrolled at public universities. The geographic distribution of university locations closely mirrored residential patterns, with most students attending institutions in the central region (*n* = 722, 55.3%). Also, undergraduate students predominated the sample (*n* = 1144, 87.7%). Academic field distribution revealed that more than half of the participants (*n* = 775, 59.4%) were enrolled in medical fields (such as medicine, dentistry, pharmacy, nursing, and allied health sciences). Table 2 depicts more details.

### 3.2. Students’ Awareness and Knowledge of Climate Change and Global Warming

**Figure 2:**
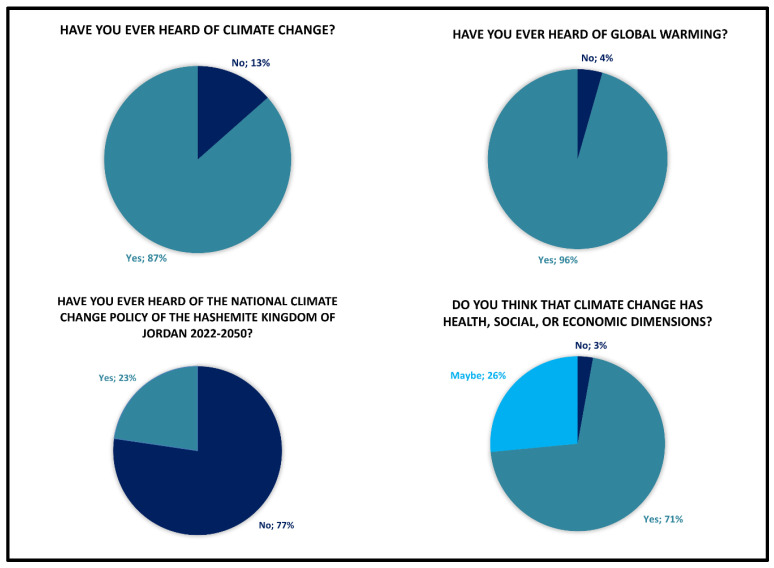
General awareness of climate change and global warming.

Figure 2 presents four key metrics assessing students’ awareness and understanding of climate change and global warming phenomena. The data revealed near-universal awareness of climate change phenomenon among students, with 87% of respondents reporting familiarity with the term climate change and 96% with global warming. Notably, 71% of respondents definitively recognize that climate change encompasses health, social, and economic dimensions. In contrast, awareness of Jordan’s national climate change policy (2022–2050) remains critically low, with only 23% of respondents reporting familiarity with this national policy.

### 3.3. Climate Change Perceptual Awareness and Climate Change Anxiety Scores

#### 3.3.1. Climate Change Perceptual Awareness Scale (CCPAS)

The overall CCPAS score demonstrated a median of 51 (IQR: 45–58). When examining individual subscales, temperature dimension yielded the highest median score of 12 (IQR: 10–13). The drought/water scarcity dimension revealed a median score of 11 (IQR: 9–12). The feelings dimension, which captures emotional and affective responses to climate change, yielded a median score of 19 (IQR: 15–23). Notably, the media dimension recorded the lowest median score of 9 (IQR: 8–12). See Table 3.

#### 3.3.2. Climate Change Anxiety Scale (CCAS)

The overall CCAS score revealed a median of 21 (IQR: 15–31), indicating mild-to-moderate levels of climate change anxiety within the sample. Analysis of subscales showed that cognitive–emotional impairment exceeded functional impairment, with medians of 12 (IQR: 9–18) and 8 (IQR: 5–12), respectively. See Table 3.

### 3.4. Comparison of CCPAS and CCAS Scores Across Participant Characteristics

CCPAS scores were significantly higher among females than males (*p* < 0.001), among students living in the family house compared with university dormitories (*p* = 0.005), and among postgraduate compared with undergraduate students (*p* = 0.034). Higher CCPAS scores were also observed among participants who had heard of climate change (*p* = 0.006) and global warming (*p* = 0.002), as well as among those who believed that climate change may affect health, society, or the economy (*p* < 0.001). No statistically significant differences in CCPAS were found according to nationality (*p* = 0.245), marital status (*p* = 0.085), residence area (*p* = 0.090), region of residence (*p* = 0.526), household size (*p* = 0.075), family income (*p* = 0.054), chronic medical conditions (*p* = 0.063), mental health conditions (*p* = 0.257), university type (*p* = 0.841), university location (*p* = 0.160), study field (*p* = 0.512), or awareness of the National Climate Change Policy of Jordan 2022–2050 (*p* = 0.922).

For CCAS, significantly higher scores were also observed among females (*p* = 0.004), students living in the family house (*p* < 0.001), those residing in rural areas (*p* = 0.019), students from the southern region (*p* < 0.001), participants from larger households (*p* < 0.001), and those with lower family income (*p* < 0.001). Additionally, higher CCAS scores were found among participants with diagnosed mental health conditions (*p* = 0.003), students in public universities (*p* < 0.001), undergraduates (*p* = 0.005), and those who had not heard of climate change (*p* < 0.001) or global warming (*p* = 0.021). No significant differences in CCAS were found by nationality (*p* = 0.988), marital status (*p* = 0.278), chronic non-mental health conditions (*p* = 0.982), university location (*p* = 0.444), study field (*p* = 0.123), awareness of the national climate change policy (*p* = 0.079), or belief in the impacts of climate change (*p* = 0.060). See Table 4.

### 3.5. Relationship Between CCAS and CCPAS Scores

**Figure 3:**
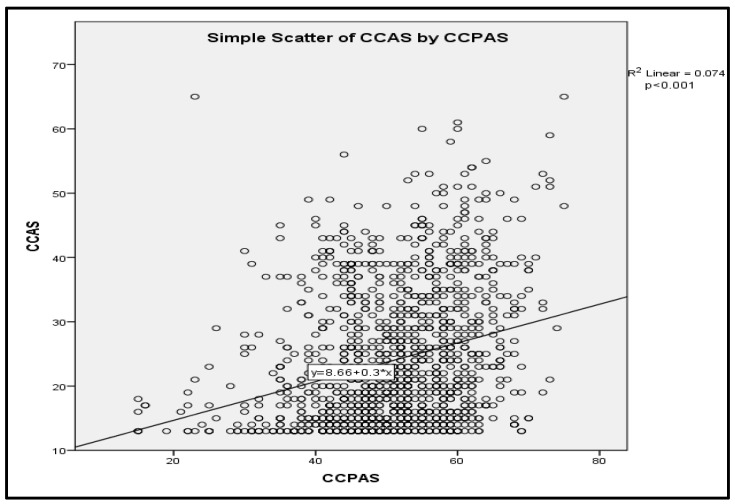
Scatter Plot of CCAS by CCPAS.

Spearman’s rank correlation showed a statistically significant positive association between CCAS and CCPAS scores (ρ = 0.276, *p* < 0.001). Additionally, in linear regression analysis, higher climate change perceptual awareness was significantly associated with higher climate change anxiety. As illustrated in Figure 3, this association was positive and statistically significant (*p* < 0.001). The fitted regression model (CCAS = 8.66 + 0.30 × CCPAS) indicates that for every one-point increase in the CCPAS score, the CCAS score increased by 0.30 points on average. However, the model explained only a small proportion of the variance in climate change anxiety (R^2^ = 0.074), indicating that factors other than perceptual awareness contributed substantially to the observed variability in anxiety.

### 3.6. Multivariable Linear Regression Analysis

In the fully adjusted multivariable linear regression models, several factors were associated with CCAS and CCPAS. For CCAS, higher scores were observed among female students compared with males (β = 1.71, 95% CI: 0.41 to 3.01, *p* = 0.010), married students compared with singles (β = 2.73, 95% CI: 0.59 to 4.88, *p* = 0.012), and those living with ≥5 household members compared with ≤4 (β = 2.85, 95% CI: 1.63 to 4.08, *p* < 0.001). Higher CCAS scores were also found among students who reported being diagnosed with a mental health condition (β = 3.95, 95% CI: 1.38 to 6.52, *p* = 0.003) and among students attending universities located in the central region of Jordan (β = 1.99, 95% CI: 0.42 to 3.55, *p* = 0.013). In contrast, lower CCAS scores were observed among students residing in the central region of Jordan (β = −1.70, 95% CI: −3.34 to −0.05, *p* = 0.043), those with a monthly income of 601–1000 Jordanian Dinars (β = −2.17, 95% CI: −3.51 to −0.83, *p* = 0.002) or >1000 Jordanian Dinars (β = −5.02, 95% CI: −6.42 to −3.63, *p* < 0.001) compared with ≤600 Jordanian Dinars, and those who had heard of climate change compared with those who had not (β = −3.68, 95% CI: −5.72 to −1.65, *p* < 0.001).

For CCPAS, higher scores were associated with female sex (β = 2.61, 95% CI: 1.30 to 3.93, *p* < 0.001), older age (β = 0.15 per year, 95% CI: 0.02 to 0.27, *p* = 0.021), and those endorsing that climate change has health, social, and economic dimensions compared with answering ‘no’ (β = 5.61, 95% CI: 0.85 to 10.37, *p* = 0.021). By contrast, lower CCPAS scores were observed among students living in university housing compared with the family house (β = −1.74, 95% CI: −3.24 to −0.23, *p* = 0.023). Overall model fit was statistically significant for both outcomes, although the explanatory power was modest and was greater for CCAS (R^2^ = 0.120; adjusted R^2^ = 0.104; *p* < 0.001) than for CCPAS (R^2^ = 0.063; adjusted R^2^ = 0.046; *p* < 0.001). Table 5 demonstrates more details.

### 3.7. Students’ Perceptions of the Impacts of Climate Change and Global Warming

**Figure 4:**
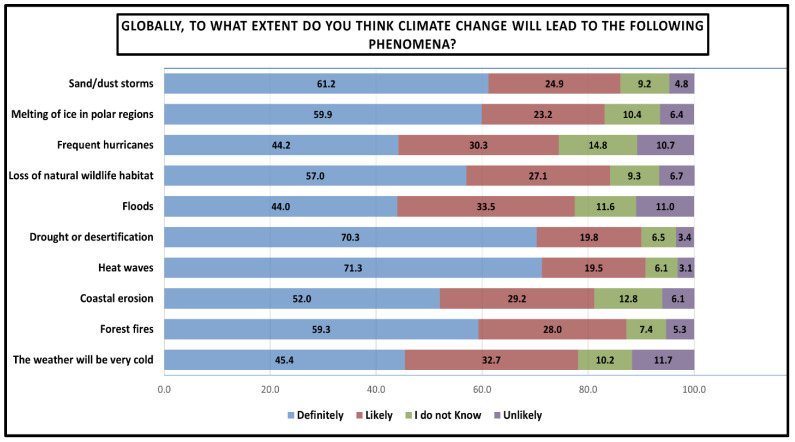
Students’ perceptions of climate change impacts across various environmental phenomena globally. Results are presented as percentages.

Figure 4 examines the respondents’ perceptions of climate change impacts worldwide across ten distinct environmental phenomena, measuring the extent to which respondents believe climate change will lead to these outcomes globally. The findings demonstrate high consensus across all phenomena, with total agreement (definitely + likely) ranging from 74.5% to 90.8%. Heatwaves showed the strongest consensus (90.8% total agreement) and notably exhibited the highest ‘definitely’ response (71.3%) among all phenomena. Droughts/desertification rank second (90.1% total agreement), while forest fires ranked the third in terms of total agreement (87.3% total agreement). Sand or dust storms follows closely (86.1% total agreement). The lowest perceived impacts, though still substantial, included frequent hurricanes (74.5% total agreement), floods (77.5% total agreement), and extreme cold events (78.1% total agreement).

**Figure 5:**
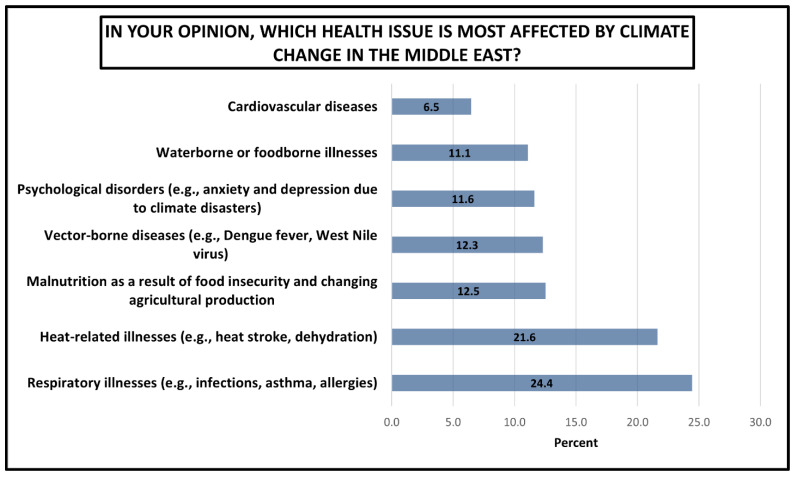
Students’ perceptions of the single most important health consequence of climate change specifically within the Middle East. Results are presented as percentages.

Regarding students’ perceptions of the single most important health consequence of climate change specifically within the Middle East context (students could choose only a single option), the results in Figure 5 reveal a clear hierarchy of perceived health vulnerabilities, with respiratory and heat-related conditions dominating student concern. Respiratory illnesses (e.g., infections, asthma, allergies) emerged as the leading health concern at 24.4%. Heat-related illnesses (e.g., heat stroke, dehydration) ranked second at 21.6%. However, the lowest perceived health impact was cardiovascular diseases (6.5%).

**Figure 6:**
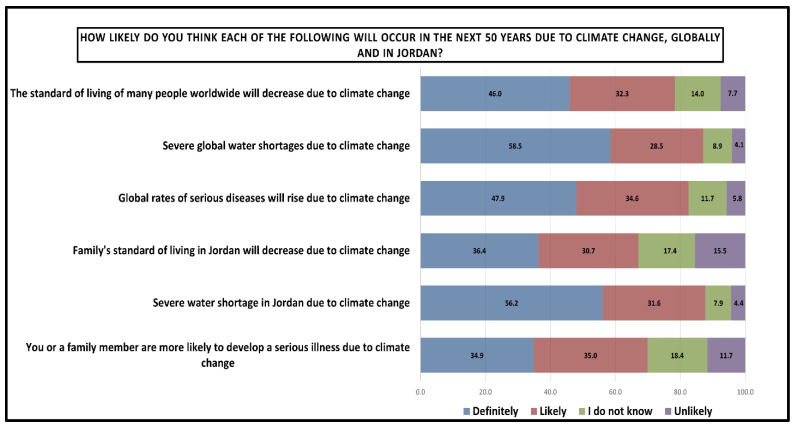
Students’ perceptions of how likely key climate change impacts are to occur over the next 50 years, comparing expected global outcomes with those anticipated specifically for Jordan. Results are presented as percentages.

Additionally, Figure 6 illustrates the students’ expectations regarding climate change impacts over the next 50 years, comparing both global and Jordan-specific outcomes as per students’ perspective. The data demonstrate high concern across all areas, with total agreement (definitely + likely) ranging from 67.1% to 87.7%. Severe water shortages in Jordan due to climate change had the strongest consensus among students (87.7% total agreement). Severe water shortage globally followed closely (87.0% total agreement). In terms of developing serious illness due to climate change, students reported a total agreement of 69.9% of having an increased vulnerability of serious illness among families in Jordan. On the other hand, reduced quality of life and standards of living among families in Jordan scored the lowest total agreement among students (67.1% total agreement) with notably the highest uncertainty and skepticism (32.9% of I do not know + unlikely).

**Figure 7:**
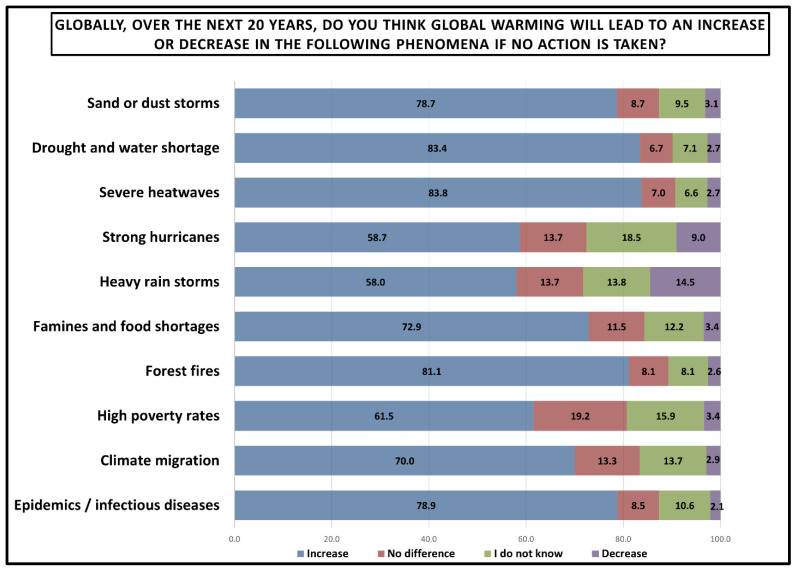
Perceived global consequences of global warming over the next 20 years if no action is taken. Results are presented as percentages.

Moreover, Figure 7 demonstrates students’ expectations regarding global warming impacts over the next 20 years if no action is taken. The data demonstrate a high expectation of increase across all phenomena, with increase responses ranging from 58.0% to 83.8%. Severe heatwaves appeared to have the strongest consensus (83.8% expect increase), while drought and water shortage followed very closely (83.4% expect increase), and forest fires ranked third (81.1% expect increase).

## 4. Discussion

The findings of this study among university students in Jordan offer a comprehensive and multidimensional understanding of how the country’s young adult population perceives climate change and experiences psychological responses linked to climate-related threats. The results reflect a pattern of high awareness of climate change and its manifestations, yet marked gaps in climate policy literacy, uneven understanding of health impacts, and a certain extent of climate-related emotional distress. A central finding in our study is the high level of general awareness of climate change, with 87% of students reporting that they have heard of the phenomenon and 96% reporting awareness of global warming. This mirrors the high baseline reported in several regional and international studies. A study in Jordan by Gazzaz et al. found that University students demonstrated a high level of knowledge regarding the nature and effects of climate change [28]. Likewise, a cross-sectional study among Al-Azhar university for girls in Egypt reported that 82.6% of students had heard of climate change and about 57.8% demonstrated good knowledge of its causes and impacts, marking climate change as a well-recognized issue across Arab youth populations [29].

Despite this widespread awareness of climate change, our study highlights a significant gap which is that only 23% of students reported familiarity with Jordan’s national climate change policy (2022–2050), revealing a disconnect between basic awareness and policy-level literacy. This gap echoes the findings of Elsharkawy et al., showing that although students expressed strong awareness of climate change in general, many were unfamiliar with the Egyptian government’s initiatives to combat climate change [29]. This disconnect between macro-level knowledge and policy-oriented literacy reinforces the need for enhanced integration of climate governance content into higher education system and public communication strategies in Jordan.

In the context of perceptual awareness, the CCPAS results in our study reveal that students demonstrate the highest perceptual sensitivity to visible climatic cues, including rising temperatures and drought/water scarcity. This pattern is closely aligned with the environmental reality of Jordan as one of the world’s most water-scarce countries; thus, it is unsurprising that students are most attuned to the threats they experience directly. Similar awareness patterns were documented among Egyptian students, who identified extreme heat, water scarcity, and desertification as the salient manifestations of climate change [29].

Our results reveal that the lowest CCPAS subscale score occurred within the media domain, suggesting limited engagement with media-based climate information. This may reflect low trust in messaging sources, inadequate tailoring of climate information for youth audiences, lack of effective framing, or oversaturation of environmental content that reduces attentiveness. Importantly, this pattern is not unique to Jordan. In a public perception study conducted across the United States, Canada, and Malta, researchers found that despite awareness of climate-health connections, climate change often lacked salience as a top-of-mind public health concern, meaning that people rarely spontaneously linked climate change with specific health risks [27]. This suggests that communication strategies across many countries struggle to translate general climate awareness into meaningful, health-focused public understanding.

In terms of climate-related anxiety, our findings show CCAS median overall score of 21, indicating mild-to-moderate levels of climate anxiety among university students in Jordan. The emotional–cognitive impairment subscale scored higher than the functional impairment subscale, implying that students experience intrusive thoughts, worry, and emotional tension about climate change, though these symptoms do not typically disrupt daily functioning. This aligns closely with the findings revealed by a recent study for validation of CCAS clinical cut-off points which demonstrated that a score of 21 represents a threshold for mild-to-moderate climate anxiety, while a score of 23 or above indicates severe or clinically concerning eco-anxiety levels [30]. However, this interpretation should be treated with caution. The CCAS is a self-reported measure, and the proposed thresholds have not yet been universally established or clinically validated across settings. Accordingly, the CCAS scores observed in our study suggest notable climate-related anxiety among students but should be interpreted as non-clinical self-reported responses rather than diagnostic evidence of a mental disorder. Additionally, eco-anxiety should not be conceptualized solely as a pathological condition. It may represent a rational and potentially adaptive response to the realities of climate change, although more intense or persistent forms may be associated with psychological distress or functional impairment in some individuals.

Furthermore, similar patterns have been documented across other settings. A study that was conducted in Istanbul, Türkiye, among nursing students demonstrated that eco-anxiety is strongly linked with anxiety, stress, and depressive symptoms, with students who believed that climate change affects them having significantly higher eco-anxiety, depression, anxiety, and stress scores [31]. Additionally, a global survey that included 10,000 children and young people globally has shown that 59% of the respondents reported being very or extremely worried about climate change, with around 45% reporting that their worry affects daily functioning, though to varying degrees depending on context [5]. Compared to these global findings, university students in Jordan appeared to experience mild-to-moderate emotional distress but lower functional impairment, implying that while climate concerns are psychologically salient, they have not yet escalated to clinically disabling distress at a large scale.

The positive association between climate change perceptual awareness and climate change anxiety suggests that students who are more aware of climate-related issues may also feel more worried or emotionally affected by them. This may be explained by the possibility that greater awareness increases recognition of the seriousness, scale, and potential consequences of climate change, which in turn may heighten concern and contribute to climate anxiety. In this sense, awareness may function as a double-edged phenomenon; while it is essential for understanding climate-related risks, it may also heighten emotional burden when individuals perceive those risks as severe or difficult to control. On the other hand, although perceptual awareness was significantly associated with climate change anxiety, the overall explanatory contribution of awareness was limited, indicating that anxiety is influenced by a much broader set of factors.

This suggests that climate change anxiety should not be viewed as merely a direct consequence of being informed. Instead, it likely arises from a complex interaction between awareness and other psychological, social, and contextual factors that were not assessed in the present analysis, such as coping capacity, perceived self-efficacy, social support, and individual personality characteristics. This distinction is important because it shows that awareness and anxiety, although related, are not interchangeable. Greater awareness may increase sensitivity to climate risks, but whether that awareness translates into anxiety likely depends on how individuals appraise the threat and whether they perceive themselves as having meaningful coping resources or avenues for action. For some students, awareness may foster concern that motivates engagement and adaptive action, whereas for others it may contribute to helplessness, uncertainty, and distress.

Moreover, the literature suggests that climate anxiety is not solely a function of climate change awareness but is shaped by broader psychosocial and contextual factors, such as perceived vulnerability, coping resources, social and cultural influences, and trust in institutions and governance [32,33,34,35,36]. Evidence suggests that even individuals who recognize the severity of climate change impacts do not always experience high psychological distress. This is particularly the case when climate risks are perceived as geographically or temporally distant, or when adaptive coping, resilience resources, and certain religious or worldview frameworks help to buffer emotional strain [35,37,38,39,40,41,42]. Together, our findings support the emerging consensus that eco-anxiety is not a simple linear function of awareness but could be mediated by broader contextual, psychological, and sociocultural factors that shape how individuals internalize climate threats. From a public health and educational perspective, this finding highlights the need for balanced climate communication. Efforts to raise awareness among university students remain important, but awareness-building alone may be insufficient and, in some cases, may inadvertently amplify distress if not accompanied by supportive strategies. Climate education should therefore be framed in a way that not only improves understanding of risks, but also strengthens resilience, promotes hope, and emphasizes practical and collective responses. Presenting climate change only as a large and overwhelming threat may increase anxiety, whereas coupling awareness with empowerment and actionable solutions may help transform concern into constructive engagement.

Several sub-group differences in climate-related anxiety were observed, including higher eco-anxiety scores among female students, married students, students from larger households, those reporting diagnosed mental health conditions, and those attending universities in the central region. Although these patterns are plausible and consistent with the possibility that social roles, household responsibilities, prior psychological vulnerability, and institutional or regional context may shape climate-related emotional responses, they should not be interpreted as definitive independent effects. Given the convenience sampling design, these associations may partly reflect the composition of the study sample, differential participation across universities or regions, and residual confounding by unmeasured factors. In particular, coping efficacy, institutional trust, direct experience with climate-related events, and exposure to climate-related media or education were not assessed and may influence both climate change awareness and anxiety. Accordingly, these sub-group findings should be regarded as exploratory, warranting confirmation in probability-based, longitudinal, and contextually richer studies. Also, the divergent associations observed for university location and residential region warrant cautious interpretation. Rather than indicating a simple geographic effect, they may reflect different dimensions of students’ lived environments, including daily campus exposure, commuting patterns, household context, and access to social support.

Our findings on students’ perceptions of environmental impacts further reinforce the depth of climate awareness among university students. Students expressed extremely high levels of agreement that climate change will cause severe global environmental disruptions, particularly through heatwaves, drought/desertification, and forest fires. These perceptions are consistent with the Intergovernmental Panel on Climate Change (IPCC) assessments, which identify the Mediterranean region as a climate change hotspot where atmospheric warming has already exceeded the global average since the 1980s and where future annual and summer warming are projected to be around 20% and 50% higher than the global annual average, respectively [43].

The health-related perceptions in our study provide an important lens from a public health perspective. Students identified respiratory illnesses and heat-related illnesses as the most important health consequences of climate change within the Middle Eastern context, while cardiovascular impacts were markedly under-recognized. This ranking reflects the epidemiological reality of the region, where heat exposure, air pollution, dust storms, and atmospheric particulate matter constitute major climate-linked health burdens. A recent study in Jordan similarly revealed high levels of awareness among medical students regarding air quality-related illnesses, heat- and cold-related conditions, vector-borne diseases, and respiratory impacts [44]; a trend echoed in public perception studies, where non-communicable diseases are met with skepticism or limited recognition, despite robust epidemiological evidence that environmental factors like heat and air pollution substantially increase cardiovascular morbidity and mortality [45,46,47,48]. This reflects a broader trend in global climate-health perception, where the public tends to focus on direct, immediate, and visually salient health threats, such as respiratory diseases, heat injury, and infectious diseases, while underestimating indirect or chronic pathways such as cardiovascular strain, mental health impacts, nutritional deficiencies, and the exacerbation of non-communicable diseases.

In the three-country survey by Akerlof et al., respondents strongly associated climate change with certain non-communicable diseases outcomes, particularly respiratory diseases and cancer, yet demonstrated far lower recognition of cardiovascular risks [27]. Moreover, classic non-communicable diseases such as obesity and heart disease were viewed as far greater direct health threats than climate change itself, reflecting a persistent gap in public understanding of indirect and long-term climate– non-communicable diseases pathways. These findings underscore the consistent need for climate-health communication efforts that highlight less visible but equally serious health impacts, particularly in regions like Jordan where pre-existing cardiovascular burdens are already high.

Additionally, our study demonstrates strong expectations of future climate impacts at both global and national levels, with majority of students expecting severe water shortages in Jordan in the next 50 years, which is an accurate reflection of Jordan’s hydrological projections, and concerns about declining quality of life and increased illnesses. These expectations are consistent with findings from other studies, where students expressed high anticipation of worsening environmental conditions and increasing resource scarcity [29,49,50,51]. Interestingly, our findings show that students exhibit a nuanced understanding of the interaction between climate change and quality of life, expressing concern not only for physical health but also for socioeconomic stability. These themes were also echoed in global studies where youth emphasized fears for future generations, concerns about government inaction, and existential distress related to perceived loss of environmental stability [5,52].

Our study findings suggest several potential implementation pathways for environmental health and climate-health promotion in university settings. Universities could integrate climate-health content into existing courses, particularly in public health, medicine, environmental sciences, and social sciences, while also offering co-curricular activities such as climate-health seminars, student-led awareness campaigns, policy dialog sessions, and community-based environmental health projects. At the policy level, national climate communication strategies could be adapted for young people by linking climate change to locally relevant health risks, such as heat-related illness, respiratory disease, water scarcity, and food security. Student wellbeing services may also consider climate-related anxiety as part of broader psychosocial support, with emphasis on coping and constructive engagement.

In terms of educational reforms, the findings emphasize the need to embed climate change across study disciplines. Climate change courses should become compulsory at faculty or university levels, ensuring that future graduates in all fields possess essential climate literacy [28]. Interdisciplinary climate-health modules should be integrated into university curricula, reflecting the multisectoral nature of climate impacts. This aligns with the recommendations of a recent study, which stresses the need to include climate-health content in medical education to prepare future health professionals for emerging challenges [44]. Also, government authorities should collaborate with higher education institutions to mainstream climate-health communication, prioritize health-centered narratives, address misinformation, and invest in climate-resilient public health systems. This recommendation is consistent with regional evidence highlighting the need to formally integrate climate content into higher education curricula [53].

Considering the broader context of public health and climate governance, the findings also demonstrate critical implications for Jordan’s national adaptation strategies. The low awareness of Jordan’s national climate change policy (2022–2050) suggests that national climate planning remains largely technocratic and insufficiently communicated to the public. As revealed by a multinational survey on public perceptions, climate change is often not cognitively linked to health outcomes without explicit cues, despite clear evidence of climate-health vulnerability [27]. Therefore, Jordanian authorities should jointly prioritize health-centered climate communication campaigns that elevate climate change as a health issue rather than an environmental or energy concern alone.

Regarding psychological and mental health implications, our findings of mild-to-moderate eco-anxiety highlight the possible need for integrating climate mental health considerations into university counseling systems, public health planning, and youth engagement programs. The CCAS clinical cut-off study emphasizes that scores around 21–23 represent meaningful psychological distress that may lead to functional impairment if environmental conditions worsen or if youth perceive ongoing governmental inaction [30]. This observation has implications for designing resilience-focused interventions, such as climate coping skill workshops, group psychoeducation, environmental volunteering programs, and collective-action engagement pathways.

Overall, our study findings indicate that university students in Jordan are not only aware of climate change but are also concerned by it and emotionally affected by its implications. However, important gaps remain in institutional communication and climate policy literacy. Addressing these gaps will be crucial for equipping the next generation of Jordan’s workforce, policymakers, and public health leaders to respond effectively to a climate-challenged future. This study adds meaningful evidence to the growing regional literature on climate change awareness and eco-anxiety, and its findings can help guide national climate adaptation efforts, educational reforms, youth engagement initiatives, and public health resilience planning.

This study has several limitations that should be considered when interpreting the findings. First, the cross-sectional observational design precludes causal inference. The observed associations between climate change perceptual awareness, climate-related anxiety, and students’ perceptions of climate impacts represent relationships measured at a single point in time and cannot establish temporality or directionality. Accordingly, our findings should be interpreted as associative and hypothesis-generating rather than as evidence that higher awareness leads to anxiety, or that anxiety shapes perceived climate risk. Similarly, the study was not designed to evaluate the effectiveness of climate-health education, youth engagement, or governance-related interventions. Any implications for strengthening climate-health literacy or youth participation in climate policy should therefore be interpreted cautiously as potential areas for future investigation. Longitudinal, interventional, and implementation-focused studies are needed to determine whether structured climate-health education, participatory youth engagement, or other institutional approaches can improve climate literacy, reduce maladaptive climate-related anxiety, and support constructive climate action among university students in Jordan. Second, the study relied on a non-probabilistic convenience sampling approach; therefore, the sample cannot be considered fully representative of all university students in Jordan, and the generalizability of the findings should be interpreted with caution. Although substantial efforts were made to engage higher education institutions across all regions of the country, the possibility of sampling bias remains. In addition, the online mode of data collection may have introduced selection bias by favoring students with reliable internet access, higher digital literacy, or greater interest in climate and environmental issues. As we were unable to determine how many students were invited and reached, participation bias could not be formally assessed. Self-selection is possible, particularly among students with prior interest in climate change, environmental issues, or public health. Third, the use of self-reported measures may have introduced recall bias and social desirability bias, particularly in relation to climate awareness and emotional responses. Fourth, differences in CCAS and CCPAS scores across participant characteristics should be interpreted cautiously, as these patterns may have been influenced by unmeasured or uncontrolled confounding and therefore do not necessarily represent independent effects. For this reason, a more conservative analytical approach was adopted. Fifth, the study did not assess potentially relevant moderating or confounding variables, such as coping efficacy, institutional trust, environmental context, or prior exposure to climate-related events, all of which may shape climate-related perceptions and anxiety. Sixth, although the quantitative instruments used were robust and appropriate for the study objectives, they did not allow for in-depth exploration of the subjective and contextual dimensions of the students’ lived experiences, which could have been better captured through qualitative inquiry. Seventh, while international studies were used for contextual comparison, differences in cultural, socio-political, and educational settings may limit direct comparability across contexts. Finally, the Arabic translation of the 15-item CCPAS demonstrated only preliminary psychometric adequacy within this university student sample. Further validation is therefore warranted in more diverse Arabic-speaking populations to assess its measurement properties. Despite these limitations, we believe that the study provides valuable baseline evidence and contributes meaningfully to the emerging climate-health literature in Jordan and the region.

## 5. Conclusions

Our study underscores a critical gap among university students in Jordan; while climate change is widely recognized and psychologically salient, awareness of the national climate change policy remains limited. These findings position climate change not only as an environmental issue, but also as an educational, psychological, and public health priority. If this generation is to contribute effectively to adaptation and resilience, awareness must be translated into informed action. Embedding climate and climate-health education across university curricula, communicating national climate strategies in accessible and youth-responsive ways, and creating formal pathways for youth participation in governance are no longer optional. They are essential investments in Jordan’s climate resilience, health security, and long-term sustainable development.

## Figures and Tables

**Table 1:** Sociodemographic characteristics of respondents (*n* = 1305).

		Frequency	Percent
**Age (years)**	**Median (IQR)**	21.0 (19.0–23.0)	
**Sex**	Male	285	21.8
	Female	1020	78.2
**Marital status**	Married	136	10.4
	Single	1169	89.6
**Nationality**	Jordanian	1179	90.3
	Non-Jordanian	126	9.7
Region of residence *	Northern region	505	38.7
	Central region	721	55.3
	Southern region	79	6.0
**Current residence place**	Residing in family house	1070	82.0
	University dormitory	235	18.0
**Current residence area**	Rural area/village	486	37.2
	Urban/city	819	62.8
**Number of household members**	≤4	484	37.1
	≥5	821	62.9
**Average family income per month ** **^+^**	≤600 JD	641	49.1
	601–1000 JD	373	28.6
	>1000 JD	291	22.3
**Currently diagnosed chronic medical conditions under treatment (non-mental health)**	No	1108	84.9
	Yes	197	15.1
**Currently diagnosed mental health conditions under treatment (e.g., anxiety disorders, mood disorders, depression)**	No	1207	92.5
	Yes	98	7.5

* Northern region (Ajloun, Irbid, Jerash, Mafraq); central region (Amman, Balqa, Madaba, Zarqa), southern region (Aqaba, Karak, Maan, Tafileh); JD: Jordanian dinar; ^+^ One JD = 1.41 U.S. Dollar.

**Table 2:** Academic profile of respondents (*n* = 1305).

		Frequency	Percent
**Classification of university/college**	Public	909	69.7
	Private	396	30.3
Location of university/college *	Northern region	509	39.0
	Central region	722	55.3
	Southern region	74	5.7
**Academic stage**	Undergraduate	1144	87.7
	Postgraduate	161	12.3
**Study field**	Medical	775	59.4
	Non-Medical	530	40.6

* Northern region (Ajloun, Irbid, Jerash, Mafraq); central region (Amman, Balqa, Madaba, Zarqa); southern region (Aqaba, Karak, Maan, Tafileh).

**Table 3:** Results of CCPAS and CCAS, including overall score and dimension’s score.

	Subscale	Median (IQR)	(Min–Max)
**Climate Change Perceptual Awareness Scale (CCPAS)**	Drought/water scarcity	11 (9–12)	(3–15)
	Media	9 (8–12)	(3–15)
	Temperature	12 (10–13)	(3–15)
	Feelings	19 (15–23)	(6–30)
	**Overall CCPAS score**	51 (45–58)	(15–75)
**Climate Change Anxiety Scale (CCAS)**	Cognitive–emotional impairment	12 (9–18)	(8–40)
	Functional impairment	8 (5–12)	(5–25)
	**Overall CCAS score**	21 (15–31)	(13–65)

**Table 4:** Comparison of CCPAS and CCAS scores across students’ characteristics.

Variables		CCPAS (Median, IQR)	*p*-Value	CCAS (Median, IQR)	*p*-Value
**Sex**	Male	49 (44–55)	**<0.001**	19 (14–29)	**0.004**
	Female	52 (45.25–58)		21 (15–31)	
**Nationality**	Jordanian	51 (45–58)	0.245	21 (15–31)	0.988
	Non-Jordanian	50 (44–56.75)		21 (15–31)	
**Marital Status**	Married	53 (46.25–57.75)	0.085	22 (15–33)	0.278
	Single	51 (45–58)		21 (15–31)	
**Current residence place**	Family house	52 (45–58)	**0.005**	21 (15–31)	**<0.001**
	University dormitory	49 (45–56)		19 (14–27)	
**Current residence area**	Rural/village	50 (44.75–57)	0.090	22 (15–32)	**0.019**
	Urban/city	51 (46–58)		20 (15–30)	
Region of residence *	Northern region	51 (45–57)	0.526	22 (16–33)	**<0.001**
	Central region	51 (45–58)		19 (15–29)	
	Southern region	51 (45–58)		23 (15–31)	
**Number of household members**	≤4	50 (45–57)	0.075	18 (14–26)	**<0.001**
	≥5	52 (45–58)		23 (16–33)	
**Average family income per month ** **^+^**	≤600 JD	50 (44–58)	0.054	23 (16–34)	**<0.001**
	601–1000 JD	52 (46–57)		20 (15–29)	
	>1000 JD	52 (47–58)		17 (14–25)	
**Currently diagnosed chronic medical conditions under treatment (non-mental health)**	No	51 (45–58)	0.063	21 (15–31)	0.982
	Yes	52 (46–58)		20 (15–30)	
**Currently diagnosed mental health conditions under treatment (e.g., anxiety disorders, mood disorders, depression).**	No	51 (45–58)	0.257	20 (15–30)	**0.003**
	Yes	51 (45.75–58.25)		24.50 (16.75–36.75)	
**Classification of university/college**	Public	51 (45–58)	0.841	21 (16–32)	**<0.001**
	Private	51 (46–57)		19 (14–28.75)	
Location of university/college *	Northern region	51 (45–57)	0.160	21 (15–31)	0.444
	Central region	51 (45.75–58)		20 (15–30)	
	Southern region	53 (46.75–58)		22.50 (15–31)	
**Academic stage**	Undergraduate	51 (45–58)	**0.034**	21 (15–31)	**0.005**
	Postgraduate	52 (47–58)		18 (14–27)	
**Study field**	Medical	51 (45–57)	0.512	20 (15–30)	0.123
	Non-Medical	51.5 (45–58)		21 (15–32)	
**Have you ever heard of climate change?**	No	49 (44–55)	**0.006**	26 (17.25–37)	**<0.001**
	Yes	51 (45–58)		20 (15–29)	
**Have you ever heard of global warming?**	No	45 (38–56.25)	**0.002**	24.50 (16–38.25)	**0.021**
	Yes	51 (45–58)		21 (15–30)	
**Have you ever heard of the National Climate Change Policy of Jordan 2022–2050?**	No	51 (45–58)	0.922	20 (15–30)	0.079
	Yes	50 (45–58)		22 (15–33.75)	
**Do you believe that climate change may impact health, society, or the economy?**	No	45 (37–54)	**<0.001**	19 (14–37.50)	0.060
	Yes	52 (46.75–58)		20 (15–30)	
	Maybe	49 (43–56)		22 (16–32)	

* Northern region (Ajloun, Irbid, Jerash, Mafraq); central region (Amman, Balqa, Madaba, Zarqa); southern region (Aqaba, Karak, Maan, Tafileh); JD: Jordanian dinar; ^†^ One JD = 1.41 U.S. Dollar. Bold values indicate *p* < 0.05.

**Table 5:** Adjusted multivariable linear regression models for CCAS and CCPAS.

Variable	Category	CCAS: β (95% CI)	*p*-Value	CCPAS: β (95% CI)	*p*-Value
**Sex**	Ref: Male	-	-	-	-
	Female	**1.71 (0.41 to 3.01)**	**0.010**	**2.61 (1.30 to 3.93)**	**<0.001**
**Age (years)**	Per 1-year increase	0.00 (−0.15 to 0.15)	0.986	**0.15 (0.02 to 0.27)**	**0.021**
**Marital status**	Ref: Single	-	-	-	-
	Married	**2.73 (0.59 to 4.88)**	**0.012**	0.23 (−1.49 to 1.95)	0.795
**Nationality**	Ref: Jordanian	-	-	-	-
	Non-Jordanian	1.11 (−0.88 to 3.11)	0.274	−0.40 (−2.40 to 1.59)	0.691
Region of residence *	Ref: Northern region	-	-	-	-
	Central region	**−1.70 (−3.34 to −0.05)**	**0.043**	−0.63 (−2.17 to 0.91)	0.424
	Southern region	−0.74 (−3.58 to 2.10)	0.608	−0.33 (−2.99 to 2.32)	0.805
**Current residence place**	Ref: Family house	-	-	-	-
	University dormitory	−1.31 (−2.86 to 0.23)	0.096	**−1.74 (−3.24 to −0.23)**	**0.023**
**Current residence area**	Ref: Urban/city	-	-	-	-
	Rural/village	−0.03 (−1.37 to 1.31)	0.964	−0.38 (−1.65 to 0.88)	0.554
**Number of household members**	Ref: ≤4	-	-	-	-
	≥5	**2.85 (1.63 to 4.08)**	**<0.001**	0.85 (-0.31 to 2.01)	0.151
**Average family income per month ** **^+^**	Ref: ≤600 JD	-	-	-	-
	601–1000 JD	**−2.17 (−3.51 to −0.83)**	**0.002**	0.05 (−1.18 to 1.28)	0.932
	>1000 JD	**−5.02 (−6.42 to −3.63)**	**<0.001**	0.80 (−0.66 to 2.26)	0.281
**Currently diagnosed chronic medical conditions under treatment (non-mental health)**	Ref: No	-	-	-	-
	Yes	0.06 (−1.63 to 1.75)	0.940	0.46 (−1.09 to 2.00)	0.561
**Currently diagnosed mental health conditions under treatment**	Ref: No	-	-	-	-
	Yes	**3.95 (1.38 to 6.52)**	**0.003**	0.37 (−1.92 to 2.65)	0.754
**Classification of university/college**	Ref: Public	-	-	-	-
	Private	−1.27 (−2.60 to 0.06)	0.062	−0.17 (−1.38 to 1.05)	0.791
Location of university/college *	Ref: Northern region	-	-	-	-
	Central region	**1.99 (0.42 to 3.55)**	**0.013**	1.35 (−0.09 to 2.79)	0.067
	Southern region	1.95 (−0.96 to 4.85)	0.190	2.53 (−0.04 to 5.09)	0.053
**Academic stage**	Ref: Undergraduate	-	-	-	-
	Postgraduate	−1.01 (−2.92 to 0.90)	0.302	0.30 (−1.41 to 2.01)	0.733
**Study field**	Ref: Non-medical	-	-	-	-
	Medical	−0.88 (−2.06 to 0.31)	0.146	−0.31 (−1.41 to 0.78)	0.574
**Have you ever heard of climate change?**	Ref: No	-	-	-	-
	Yes	**−3.68 (−5.72 to −1.65)**	**<0.001**	0.04 (−1.54 to 1.63)	0.956
**Have you ever heard of global warming?**	Ref: No	-	-	-	-
	Yes	−2.80 (−6.39 to 0.80)	0.127	3.18 (−0.49 to 6.85)	0.089
**Have you ever heard of the National Climate Change Policy of Jordan 2022–2050?**	Ref: No	-	-	-	-
	Yes	1.32 (−0.07 to 2.70)	0.062	0.06 (−1.17 to 1.28)	0.927
**Do you believe that climate change may impact health, society, or the economy?**	Ref: No	-	-	-	-
	Maybe	−0.63 (−5.18 to 3.92)	0.787	3.10 (−1.77 to 7.96)	0.212
	Yes	0.03 (−4.44 to 4.49)	0.991	**5.61 (0.85 to 10.37)**	**0.021**

* Northern region (Ajloun, Irbid, Jerash, Mafraq); central region (Amman, Balqa, Madaba, Zarqa); southern region (Aqaba, Karak, Maan, Tafileh); JD: Jordanian dinar; ^+^ One JD = 1.41 U.S. Dollar. Bold values indicate *p* < 0.05. Ref: Reference category.

## Data Availability

The dataset generated and analyzed during the current study is not publicly available due to institutional restrictions. De-identified data may be made available from the corresponding author on a reasonable request and following approval from the Jordan Center for Disease Control and relevant ethics committee. Additionally, the survey instrument used in this study forms part of a broader climate change research project in Jordan led by the Jordan Center for Disease Control. The 15-item Climate Change Perceptual Awareness Scale and the 13-item Climate Change Anxiety Scale are publicly available instruments, and their original sources are cited in the manuscript. Other study-specific questionnaire items may be made available to interested researchers upon reasonable request directed to Jordan Center for Disease Control, subject to applicable institutional approvals and data governance procedures.

## Abbreviations

MENA	Middle East and North Africa
CCPAS	Climate Change Perceptual Awareness Scale
CCAS	Climate Change Anxiety Scale
JCDC	Jordan Center for Disease Control
IPCC	Intergovernmental Panel on Climate Change
IQR	Interquartile Range
SD	Standard Deviation
